# Restoring and maintaining robust maternity services in the COVID-19 era: a public health dilemma in Zimbabwe

**DOI:** 10.11604/pamj.supp.2020.37.1.26798

**Published:** 2020-11-08

**Authors:** Grant Murewanhema, Munyaradzi Innocent Nyakanda, Mugove Gerald Madziyire

**Affiliations:** 1 Unit of Obstetrics and Gynaecology, Department of Primary Health Care Sciences, Faculty of Medicine and Health Sciences, University of Zimbabwe, Harare, Zimbabwe,; 2 Zimbabwe Society of Obstetricians and Gynaecologists, Harare, Zimbabwe,; 3 Sally Mugabe Hospital, Ministry of Health and Child Care, Harare, Zimbabwe

**Keywords:** COVID-19, Zimbabwe, delays, maternity services, essential health services

## Abstract

Lockdown policies, travel restrictions and reduced provision of healthcare in Zimbabwe in response to the COVID-19 pandemic have brought unprecedented challenges for healthcare delivery. Maternity services, including antenatal care, labour and delivery as well as postnatal care have been affected directly and indirectly by the pandemic and resultant control interventions, with delays introduced at several points across the continuum of care. Unfortunately, maternity conditions are time-sensitive, and delays can negatively impact feto-maternal outcomes, with increased maternal, fetal or neonatal morbidity and mortality. An audit at central hospitals revealed reduced utilisation of maternity services and a trend towards an increase in maternal mortality. A formal evaluation is required; however, mitigating public health interventions are required, especially as the burden of COVID-19 in the country has considerably come down. The World Health Organisation offers useful technical guidance for maintaining essential health services in pandemic times in low-resources settings, and rationalising the use of personal protective equipment, which can be contextualised and adopted to restore and maintain essential health services. Restoration of essential maternity services is urgently required in an environment that protects healthcare workers and their clients, minimising their risk of contracting COVID-19 whilst optimising fetomaternal outcomes. Thus, the various stakeholders involved in maternity care must urgently come together and find ways of achieving this goal.

## Perspectives

Zimbabwe recorded its first case of COVID-19 in March 2020 [1]. The government responded by introducing lockdown policies and travel restrictions to curb the further spread of SARS-CoV-2, the causative virus for COVID-19, prepare the health system for an upsurge of cases, and mitigate against the damage from the outbreak [2]. The public health sector restricted service provision to emergency mode, effectively stopping all non-emergency care including outpatient care and elective surgeries [2]. Though service provision is slowly returning to considerably normal levels, the major hospitals are still operating in a semi-emergency mode. Supply chain disruptions introduced by international and local travel restrictions have resulted in shortages of essential medicines and personal protective equipment (PPE), imported by Zimbabwe and other developing countries [3].

A critical area of healthcare provision is maternity services, which includes antenatal care (ANC), labour and delivery and postnatal care (PNC). Pregnancy is a time-sensitive condition, and delays in care and service provision can dramatically precipitate undesirable maternal, fetal or placental sequelae, leading to maternal, fetal or infant death or morbidity. The leading direct causes of maternal mortality (MM) in the developing world including Zimbabwe, namely hypertensive disorders of pregnancy, post-partum haemorrhage and sepsis [4] are all time-sensitive, and delays introduced by the COVID-19 pandemic can worsen outcomes. Zimbabwe is one of the countries with the highest maternal mortality rates (MMRs) in the world at 614 deaths per 100 000 live births [5]. It is still a long way from achieving Sustainable Development Goal 3 (SDG3), which aims to reduce the average global MMR to less than 70 deaths per 100 000 women, leaving no country with an MMR greater than double the global average [6]. Maternal health surveillance may have been weakened by the COVID-19 restrictions. However, an unpublished report by the Permanent Secretary in the Ministry of Health and Child Care (MOHCC) suggested a reduction in the utilisation of maternity services. This was further substantiated by an unpublished audit of maternal outcomes at two tertiary institutions in the country, which additionally revealed a trend towards a rise in MM [7]. A formal evaluation to estimate the true collateral damage on maternity outcomes stemming indirectly from the COVID-19 pandemic is warranted urgently; however, stakeholders must already expedite introducing measures to mitigate against further undesirable maternal outcomes. The aim of this paper is to conceptualise and discuss the delays, and offer possible recommendations for reintroducing and maintaining robust maternity services, and prevent further undesirable maternal outcomes.

**Conceptualising the delays:** based on Thaddeus and Maine´s conceptual framework for categorising causes of MM [8], delays are notable across the whole spectrum of maternity care, with a break in the continuum of care from ANC through to PNC [9]. First stage delays are occurring due to failure to access ANC services, where treatable or preventable pregnancy complications are usually first identified. From primary health care clinics to tertiary institutions, services were disrupted because of stoppage of routine non-emergency services, fear of contracting COVID-19 among healthcare workers (HCWs), anxiety and lack of PPE [10]. Studies from other settings have shown heightened fear among HCWs [11]. Frontline workers considerably contracted and succumbed to COVID-19 in other countries. Zimbabwe recently established a fetal medicine unit, and anecdotal evidence suggests a reduction in the number of scanned pregnant mothers since the COVID-19 restrictions were introduced. Travel restrictions and shortages of public transport have also served as barriers preventing women from accessing essential maternity care services [12]. Public transport not belonging or affiliated to the national passenger carrier was banned. Earlier on in the pandemic, it was essential to provide proof of reason to travel as part of lockdown rules. Thus, even when appropriately referred, second stage delays may also occur as women fail to reach facilities timeously. At the referral facilities, several challenges exist, which introduce third stage delays. Some of these may have been pre-existing but aggravated by the COVID-19 pandemic. These centre around shortage of medicines, PPE, sundries and human resources, due to supply chain disruptions, fear and anxiety [13]. Isolating infected HCWs and quarantining the exposed may have exacerbated pre-existing staff shortages, indirectly contributing to poorer maternity outcomes.

Critical factors around HCW availability including remuneration, risk-allowances and PPE were not adequately addressed, leading to an industrial action by the frontline workers, possibly confounding the outcomes [10]. Shortages of life-saving blood products were reported in the pandemic owing to shortage of essential reagents, and a shortage of blood donors due to school closures and travel restrictions [14]. Students are the most frequent blood donors in Zimbabwe. Delayed availability of results from blood assays for haematology and biochemistry also delayed operative interventions for patients. There are reports of patients who were denied access to emergency hospital admissions because they had no test results for SARS-CoV-2 [15]. Unfortunately, reagents for testing are not always available in the public sector [13], and polymerase chain reaction (PCR) testing in the private sector is expensive [15], and beyond the reach of many, in a country where the majority are not formally employed.

**Restoration and maintenance of maternity services:** the latest situation reports from the MOHCC indicate that from the peak in July-August 2020, where the daily numbers of reported cases were in excess of 100, the incident cases have considerably dropped, and the 7-day rolling average for new cases has dropped to an average of 20, with minimal fatalities [16]. Whilst this does not necessarily signify the end of the COVID-19 era in Zimbabwe, and further waves are possible, restriction of access to maternity care may currently be doing more harm than COVID-19. Thus, urgent restoration of full maternity services is warranted, in an environment that is protective to both HCWs and clients. The World Health Organisation (WHO) provides critical guidance on how to maintain essential health services during the COVID-19 era [17]; however, the provided guidance must be contextualised. Critical to restoration and maintenance of robust maternity services is understanding the current infection dynamics in the different parts of the country, to facilitate introduction of differentiated models of prevention, based on local epidemiology.

The basics of infection prevention and control (IPC) must be upheld at all times, from community health facilities, primary health care facilities through first level hospitals to tertiary institutions. Universally in Zimbabwe this includes basic hand hygiene at all facilities with hand-washing or sanitisation ports, temperature checks, mandatory wearing of facemasks and physical distancing. Thus, facilities must continuously engage with Risk Communication and Community Engagement (RCCE) pillars to provide continuous information, education and communication (IEC) material in its various forms and in the different local languages. Risk perception challenges must be addressed adequately. Providing frontline HCWs with appropriate and adequate PPE for certain tasks is a high priority, and again the WHO offers useful technical guidance on rationale use of PPE in the pandemic in resource-limited settings [18]. Continued on-the-job training in IPC is critical for all HCWs, including midwives, doctors and all involved in maternity care. Innovative ways of keeping ANCs and PNCs decongested and maintaining physical distancing are required. These must include a strict booking system, only to be breached if client has an emergency, and a reminder system for clients to keep their appointments. Low-risk pregnancies as assessed by midwives and obstetricians must be allowed the minimum number of visits in the third trimester, but higher risk pregnancies can be allowed more visits for closer feto-maternal surveillance. The use of telemedicine, which has been shown to be a feasible option for triaging obstetric patients in the pandemic [19,20], and other digital health platforms, must be promoted. Where possible, ultrasound scan facilities must be located in the ANC, to avoid clients having to visit another facility for a scan.

In a low-resource setting, SARS-CoV-2 PCR testing is not possible for every client, and thus may need to be offered based on clinical need. However, HCWs must observe IPC precautions at all times, to protect themselves from being infected and infecting their clients. Promoting physical distancing is not just client-client, but also HCW-client, including times of examination and checking vital parameters. In the labour ward, LW, clinicians must be provided with hand-held Doppler machines to avoid auscultating with Pinard fetal stethoscopes. Table 1 lists some recommendations for restoring and maintaining essential maternity services in the COVID-19 era. In this article, we have concentrated on the mother, but the care of the newborn must be considered for complete maternity care. A multidisciplinary approach, especially around labour and delivery, between obstetricians, midwives, anaesthesiologists and paediatricians must be encouraged to optimise maternal and fetal outcomes. However, wherever possible, telemedicine must be used, even video conferencing, to discuss cases requiring multidisciplinary approaches.

**Table 1:** recommendations for restoring and maintaining essential maternity services during the COVID-19 pandemic

Component of service	Measures to ensure safe service delivery
Antenatal care	⦁ Stratify pregnancy risk:
	‣ More frequent visits for high-risk pregnancies.
	‣ Third trimester visits for low-risk pregnancies
	⦁ Offer extended doses of ferrous sulphate/folate of three-four months.
	⦁ ; Provide all relevant client care in a single visit to avoid unnecessary repeat visits
	⦁ Promote the use of telemedicine and digital platforms wherever possible. Advocacy for internet subsidies for maternity care is needed.
	⦁ Utilise the first contact fully for any missed opportunities including vaccinations and laboratory evaluations, and where possible, provide laboratory results over the phone.
	⦁ Restrict antenatal admissions to only those unavoidable, and limit the allowable number of visitors in the antenatal wards to only one appropriately screened individual.
Labour and delivery	⦁ Universal infection prevention and control measures throughout labour and delivery
	⦁ Allow only birth companion, preferably the partner, who should be appropriately screened for COVID-19.
	⦁ Caesarean sections must be performed for obstetric indications, regardless of the COVID-19 status of the patient.
	⦁ All patients requiring emergency surgery must be offered, with or without COVID-19 test results, observing universal IPC measures persistently, consistently and correctly.
	⦁ Minimise waiting times, operation and recovery times to the best possible, ensuring that the most skilled manpower is available.
	⦁ Work closely with paediatricians to urgently resuscitate babies, and shorten the time to transfer them to neonatal units.
	⦁ Whenever possible, utilise maternal waiting shelters, ensuring the most possible IPC standards are observed.
Postnatal care	⦁ Shorten the hospital duration as much as possible for patients who deliver both normally and vaginally.
	⦁ Educate patients on danger signs, and offer telephone numbers for teleconsultations.
	⦁ Where physical postnatal visits are unavoidable, offer the maximum possible care in one visit to avoid all avoidable repeat visits.

## Conclusion

Maternity services are essential health services, even during the COVID-19 pandemic. Safe and effective means of restoring and maintaining robust maternity care in a pandemic setting are urgently needed. Thus, all relevant stakeholders in maternal health must urgently convene, recommend restoration of maternity services and design effective means of doing so whilst fully protecting HCWs and clients from contracting COVID-19.
